# Is hands-on learning still necessary in the age of AI? A thematic review

**DOI:** 10.3389/fpsyg.2026.1897168

**Published:** 2026-09-09

**Authors:** Jie Yu, Li Liu, Qiuxia Zhu

**Affiliations:** 1 Faculty of Art, Lanzhou University of Finance and Economics, Lanzhou, China; 2 City University Malaysia, Petaling Jaya, Malaysia; 3 Lanzhou Jiaotong University, Lanzhou, China

**Keywords:** artificial intelligence, creative thinking, design education, design literacy, hands-on learning, human–AI collaboration, reflective learning

## Abstract

**Introduction:**

Generative artificial intelligence (AI) is increasingly entering design education and influencing how students engage in the design process. Existing studies have widely explored the potential of AI for creative production and improving design efficiency. Less attention has been paid to whether hands-on learning is still necessary in the age of AI.

**Methods:**

This study adopted a thematic review approach to examine changes in the role of hands-on learning in AI-supported design education and its educational value. The review was based on 32 peer-reviewed studies retrieved from Web of Science and Scopus.

**Results:**

The reviewed literature suggests that AI does not simply replace hands-on learning but changes how it may take place. Two interrelated forms can be distinguished: Embodied Hands-on, based on the body, tools, and materials, and Cognitive Hands-on, based on language, judgement, and human -AI iteration. Both share a cycle of action, feedback, reflection, and refinement, but their educational roles are not equivalent. The review also identifies concerns including the compression of learning processes, dependence on AI-generated outcomes, and an increasing emphasis on evaluation.

**Discussion:**

This study proposes a process-oriented framework for understanding how Embodied Hands-on and Cognitive Hands-on may relate to creative thinking, design judgement, and student agency. The findings suggest that design literacy may increasingly involve critical evaluation, metacognitive regulation, and decision-making in human -AI collaboration.

## 1 Introduction

Design education is undergoing rapid change as generative artificial intelligence (AI) becomes increasingly embedded in the design process. These tools are increasingly used to expand creative possibilities and support new forms of human–AI collaboration (Kasneci et al., 2023; Rudolph et al., 2023). Their growing use has also raised new educational questions. Design education has traditionally emphasised learning through making, iteration, critique, and reflection, while AI can now generate and modify visual alternatives with considerably less direct physical engagement.

Hands-on practice has deep historical roots in design education. Studio pedagogy has long been a defining feature that distinguishes design from many other disciplines. Students develop design judgement through continuous making, critique, and revision rather than through instruction alone (Cuff, 1991; Lawson, 2018). This tradition also has a theoretical basis. Hands-on learning is not an independent learning theory but is closely related to constructivism, experiential learning, and reflective practice. Learners construct knowledge through active engagement (Piaget, 1952). Concrete experience develops into understanding through reflection and renewed practice (Kolb, 1984). Learners also adjust their actions and judgements in response to feedback from materials and tools (Schön, 1983). Hands-on learning in its traditional sense is therefore more than making by hand. It involves a continuing process of action, feedback, reflection, and refinement. In design education, this commonly takes the form of sketching, material exploration, model making, prototype development, and repeated revision. These practices have long contributed to the development of design thinking (Cross, 1982).

This process-oriented understanding raises an important question in AI-supported design education. If hands-on learning is defined only by physical manipulation, many AI-mediated activities would fall outside its conceptual boundaries. If it is understood through the processes of action, feedback, reflection, and refinement, however, some forms of AI-supported interaction may retain similar process characteristics even when direct material manipulation is reduced. This review therefore does not assume that cognitive or AI-mediated activity is itself a form of hands-on learning. Instead, it examines under what conditions such activities may reproduce or extend some of the process characteristics traditionally associated with hands-on learning.

Generative AI is changing how students act within design learning. Students can rapidly generate and modify visual alternatives through text prompts, improving the efficiency of idea generation and expanding the scope of creative exploration (Baltà-Salvador et al., 2026; Georgieva and Georgiev, 2025). At the same time, rapid generation may reduce or reorganise processes previously carried out through sketching, material experimentation, and repeated trial and error. When parts of the design process are transferred to automated tools, opportunities for active reflection and independent judgement may also change (Risko and Gilbert, 2016; Kasneci et al., 2023). Sketches and other external representations are not used only to present outcomes; they also contribute to the generation, discovery, and reorganisation of ideas (Goldschmidt, 1991; Suwa and Tversky, 1997). Changes in these processes may therefore have implications for how students develop creative thinking and design judgement.

Existing research on AI-supported design education has largely focused on design efficiency, creative output, tool use, and human–AI collaboration. Less attention has been given to how the learning process itself is being reorganised. Relevant studies discuss physical making, prompting, comparison of generated alternatives, reflection, judgement, and human–AI interaction, but these activities are often examined separately. It therefore remains unclear how different forms of learner action and feedback relate to one another, whether AI-supported interaction can retain the process characteristics associated with hands-on learning, and how rapid generation may alter the role of iteration, trial and error, and reflection.

To address these questions, this study adopts a thematic review approach to examine literature on hands-on learning and AI-supported design education. Rather than assuming that AI-mediated activity constitutes a new form of hands-on learning, the review compares recurring forms of learner action, feedback, reflection, and revision across the selected literature. Particular attention is given to the relationship between embodied forms of practice and emerging forms of cognitive and evaluative interaction. Through this analysis, the study explores whether the conceptual boundaries of hands-on learning may be changing under AI-supported conditions and what educational tensions may arise from this change.

This study addresses the following research questions:

1. What forms of learner action, feedback, and iteration are described in AI-supported design education?
2. How do embodied practice and AI-mediated cognitive interaction relate to the process characteristics traditionally associated with hands-on learning?
3. How does rapid AI generation appear to change iterative and reflective processes in design learning, and what pedagogical tensions emerge from these changes?

## 2 Methods and procedures

### 2.1 Review approach

This study adopted a thematic review approach to identify recurring patterns, conceptual connections, and pedagogical issues related to hands-on learning practices and learning processes in AI-supported design education. As the relevant studies span different design fields, teaching contexts, research designs, and theoretical perspectives, this review does not primarily focus on effect-size calculation or bibliometric analysis. Instead, it uses cross-study comparison and thematic interpretation to develop an integrated understanding of how hands-on learning is changing with the introduction of AI.

This study focuses on learners’ process-oriented participation in design learning and on how such processes are manifested through physical making, AI-mediated interaction, and human–AI collaboration. The specific processes of coding, comparison, and theme development are described further in Section 2.4.

To improve the transparency of the review process, this study retained a clear procedure for literature identification, screening, and corpus construction. The relevant principles of PRISMA 2020 (Page et al., 2021) were used primarily to support transparent reporting of the literature identification and screening process.

### 2.2 Literature identification and search strategy

This study primarily identified relevant literature through Web of Science and Scopus to establish a review corpus covering design education, practice-based learning, and AI-supported design learning. These two databases were selected because they include peer-reviewed research across education, design, the social sciences, and related interdisciplinary fields, while also offering a degree of complementarity in coverage.

Given the long-standing development of hands-on learning in design education and the more recent changes introduced by Generative Artificial Intelligence (AI), two interconnected search pathways were used. The first focused on established traditions of practice-based and studio learning in design education, including processes such as making, prototyping, critique, iteration, and reflection. The second focused on emerging learning practices and human–AI interaction following the introduction of AI into design education. Different time ranges were used to reflect the development of these two areas. The first pathway covered 2005–2020 to identify relevant practice-based learning research established before generative AI became widely introduced into design education. The second covered 2019–2026 to reflect the rapid growth of research on AI-supported design learning in recent years.

Keyword combinations were developed around these two search pathways. The search terms mainly covered concepts related to studio pedagogy practice-based design learning design education generative AI AI-assisted design and human–AI collaboration. Specific keyword combinations were adjusted appropriately across databases according to their search fields and the results of the initial searches. The full databases search fields search strings and time ranges are presented in Table 1.

**Table 1:** Literature identification and search strategy.

Database	Search field	Search strings	Time range	Initial results (n)	Retained records (n)
Web of Science	Topic (TS)	(“studio pedagogy” OR “design studio pedagogy” OR “studio-based learning”) AND (“design education” OR “visual communication” OR “art education”)	2005–2020	38	10
Web of Science	Topic (TS)	(“design education” OR “design pedagogy” OR “graphic design education” OR “visual communication education”) AND (“generative AI” OR “artificial intelligence” OR “AI-assisted design” OR “text-to-image” OR “human–AI co-creation”)	2019–2026	22	16
Scopus	TITLE-ABS-KEY	TITLE-ABS-KEY((“design studio” OR “studio pedagogy” OR “studio-based learning”) AND (“generative AI” OR “artificial intelligence”) AND (“higher education” OR university))	2019–2026	17	10

The three principal searches yielded 36 records after initial relevance screening. After removing four duplicate records across search pathways, 32 studies were included in the final review corpus.

In Web of Science, searches were conducted mainly through the Topic (TS) field, which covers titles, abstracts, author keywords, and Keywords Plus. In Scopus, searches were conducted through the TITLE-ABS-KEY field. The literature search began in mid-March 2026 and was refined over approximately one week. Initial searches showed that different studies used different terms to describe similar learning practices and AI-supported design processes, and some relevant studies did not explicitly use the term “hands-on learning.” The keyword combinations were therefore adjusted after reviewing the titles, abstracts, and keywords of the initial results to improve the conceptual coverage of relevant learning processes. These adjustments did not change the research questions or the basic scope of study inclusion.

ERIC was also explored during the initial scoping stage. Searches using similar core concepts identified few additional studies directly addressing the intersection of hands-on learning, AI, and design education, and there was overlap with results identified through Web of Science and Scopus. ERIC was therefore not used as a primary database for the final literature identification. Relying mainly on Web of Science and Scopus may have affected the comprehensiveness of the review, and this limitation is discussed further in the limitations section.

### 2.3 Study selection and review corpus

To ensure that the included studies could address the research questions, clear inclusion and exclusion criteria were established. Eligible studies were required to be peer-reviewed publications in English, primarily journal articles and peer-reviewed conference papers with a complete research design, sufficient methodological detail, and clearly reported findings. The study context also needed to relate to design education, design learning, or practice-based design pedagogy, with substantive discussion of learning processes such as hands-on learning, making, critique, reflection, creative cognition, AI-supported learning, or human–AI collaboration.

Studies were not included or excluded solely on the basis of disciplinary labels, but according to whether they addressed design education and learning processes relevant to the research questions. Studies from architecture and other adjacent design fields were included when their main focus was design education, teaching practice, or learning processes. Studies were excluded when they focused primarily on professional practice, technical development, engineering applications, environmental performance, or algorithmic performance without substantive discussion of education or learning. As AI-supported design education is still a rapidly developing area, peer-reviewed conference papers were not excluded as a category. However, only those with a complete research design, sufficient methodological detail, and clearly reported empirical findings were retained. Conference publications available only as abstracts, studies with incomplete reporting of methods or findings, editorials, book reviews, non-peer-reviewed publications, and duplicate records were excluded.

**Figure 1:**
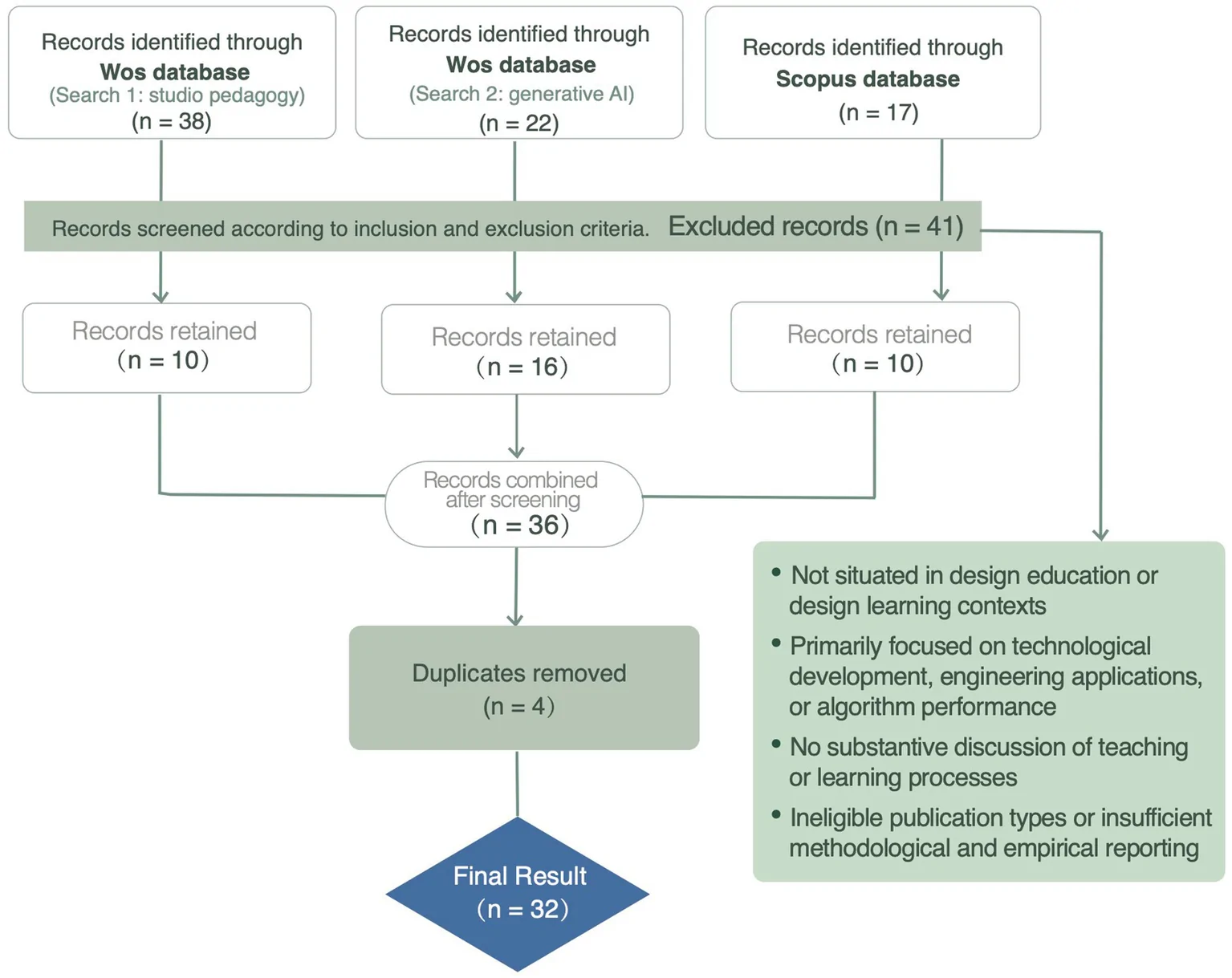
Literature identification and selection process.

Three principal searches identified 77 initial records. An initial relevance screening was then conducted within each search, mainly on the basis of titles, abstracts, and keywords, resulting in 36 retained records. Four duplicate records across the search pathways were subsequently removed, producing a final review corpus of 32 independent studies. The literature identification and selection process is shown in Figure 1.

After screening, the included studies underwent a further quality review to determine whether they provided sufficiently clear and credible methodological and empirical information to support the subsequent thematic analysis. The review drew on the appraisal principles of the Mixed Methods Appraisal Tool (MMAT; Hong et al., 2018). The quality review was conducted primarily by the first author, with the third author reviewing a subset of the appraisal results. Where uncertainty arose, the relevant authors discussed the original study in relation to the appraisal criteria before reaching a final decision.

**Figure 2:**
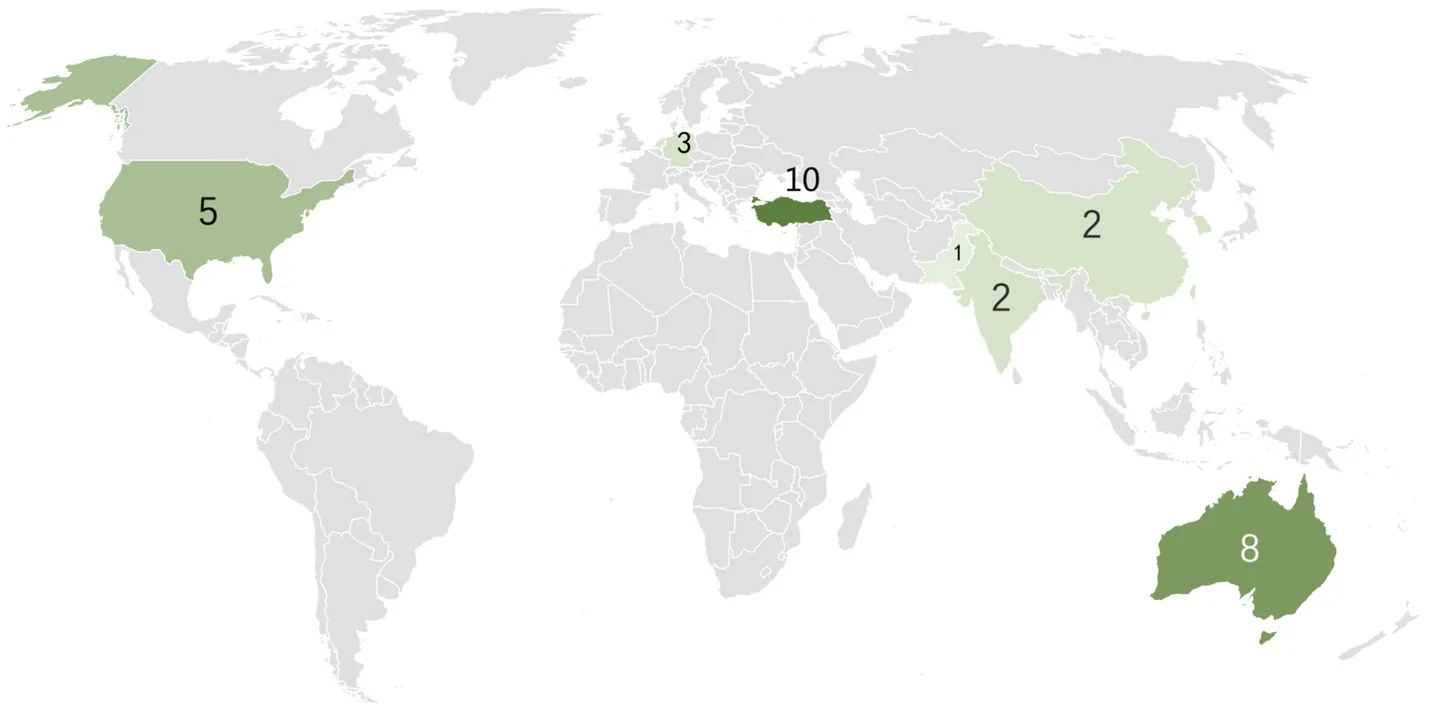
Geographical distribution of the review corpus. Regions were coded based on the research context and educational setting described in each study, rather than the authors’ institutional affiliations.

**Figure 3:**
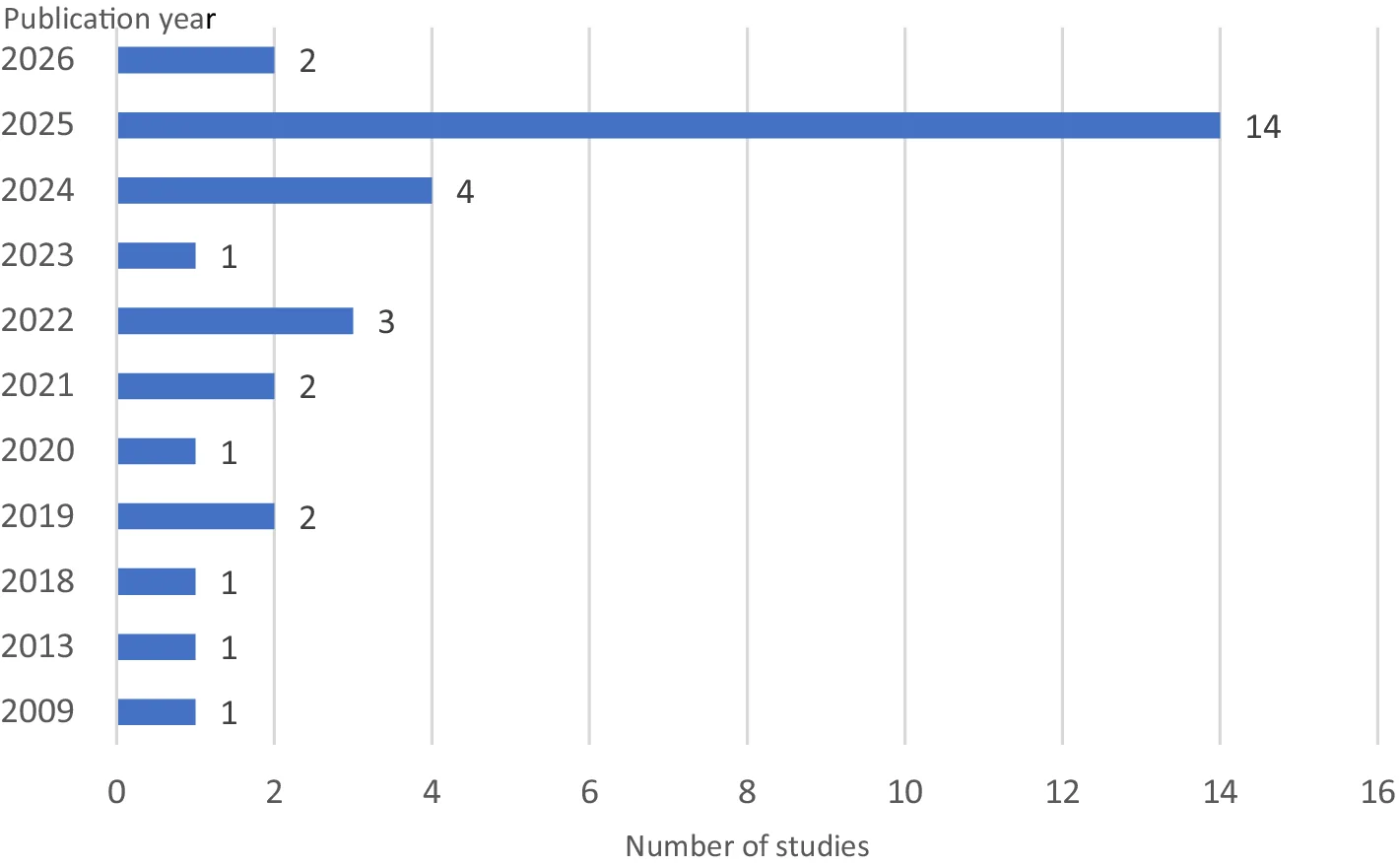
Temporal distribution of the review corpus.

The final review corpus includes both earlier research on practice-based learning in design education and more recent studies of AI-supported design education, providing a basis for subsequent thematic comparison and analysis. Figures 2, 3 present the geographical and temporal distributions of the final review corpus, respectively. Geographical classification was based on the educational context described in each study rather than the authors’ institutional affiliations, because the focus of this review is on the contexts in which teaching and learning occurred rather than on the national distribution of research output.

### 2.4 Thematic analysis and theme development

This study employed an inductive thematic analysis to code and compare the final set of included studies, with the aim of identifying recurring learning activities, cognitive processes, and pedagogical issues in AI-supported design education. The analysis was informed by the principles of thematic analysis proposed by Braun and Clarke (2006). Themes were not predetermined, but developed progressively through repeated reading, coding, comparison, and refinement. All literature management and coding were conducted using ATLAS.ti 25.

The analysis involved three stages: open coding, focused coding, and theme development. During open coding, the first author read each included study and generated initial codes related to hands-on learning and AI-supported learning processes. The coding remained as close as possible to the learning activities, interaction processes, and learner responses described in the original studies, rather than assigning data to predetermined final themes. The third author then reviewed a subset of the coded material, code definitions, and the preliminary coding framework, with particular attention to the consistency between the codes and the original studies, and contributed to refining the coding structure.

During focused coding, conceptually similar initial codes were continuously compared, filtered, and grouped to identify their shared characteristics, differences, and relationships. The purpose of this stage was not to establish a fixed intermediate category structure, but to develop higher-level thematic patterns through cross-study comparison. Content that diverged from the main patterns or presented different findings was also compared to avoid constructing themes solely from evidence pointing in one direction.

During theme development, the grouped codes were repeatedly compared with the original studies, coding materials, and research questions, and were further integrated and abstracted according to their shared process characteristics. The distinction between Embodied Hands-on and Cognitive Hands-on gradually emerged through this process of comparison and grouping rather than being predetermined at the beginning of the analysis. Embodied Hands-on developed primarily from codes related to direct making, bodily engagement, and material feedback. Cognitive Hands-on developed from codes related to activities such as generation, comparison, judgement, selection, and revision in AI-mediated learning processes. Although the two themes represent different forms of learning, both reflect sustained learner participation and continued adjustment in response to feedback.

Codes related to design literacy, learner agency, and pedagogical tensions were used to further understand the educational significance and possible limitations associated with these themes. After the themes had been developed, the researchers returned to the original studies to check whether they adequately represented recurring patterns across the literature while preserving important differences.

When disagreements arose regarding code interpretation or theme allocation, the researchers revisited the relevant source material and discussed the issue in relation to the code definitions, study content, and research questions until agreement was reached. The study used first-author-led coding with partial review by the third author rather than full independent parallel coding across the entire corpus. Statistical inter-coder reliability was therefore not calculated. The credibility and traceability of the analysis were supported through third-author review, repeated reference to the original studies, discussion of coding differences, and supplementary coding materials.

To further illustrate the process of theme development, Table 2 presents representative coding examples showing the analytical pathway from source material to initial codes and then to developed themes. A complete overview of the included studies and their analytical categories is provided in Supplementary Table S1. The overall relationship between codes and final themes is presented in Supplementary Figure S1.

**Table 2:** Illustrative examples of coding and theme development.

Source evidence/meaning unit	Initial code	Developed theme
Yorgancıoğlu and Genel (2022): sketch modelling as an iterative process based on continuous feedback from learning by doing	Embodied Engagement	Embodied Hands-on
Kavakoğlu et al. (2022): students learn through sketching in a “see–do–see loop”		
Karadağ and Ozar (2025): progressive prompting provides a basis for iterative refinement	Prompt Engineering as Practice	Cognitive Hands-on
Zhu et al. (2025a): prompt behaviours involve generation, modification and selection		

The examples shown represent selected coding pathways from the full analytical framework. The complete coding structure is presented in Supplementary Figure S1.

## 3 Findings

### 3.1 Continuation of embodied hands-on

The included studies show that sketching, model making, material experimentation, and prototype development remain important learning activities in design education. Students gradually develop design ideas through drawing, making, observing, and revising. Akalın and Sezal (2009) noted that conceptual and physical models help students observe and evaluate their own ideas. Yorgancıoğlu and Genel (2022) also found that sketch models serve exploratory, communicative, and evaluative functions. Together, these studies show that Embodied Hands-on is not used only to present design outcomes. It also contributes to the development, testing, and refinement of ideas.

Before the emergence of Generative Artificial Intelligence (AI), digital technologies had already begun to change practice in design education. Oxman (2008) noted that digital design introduced not only new representational tools but also changes in how design knowledge and methods were formed. Hardman (2022) described this as part of the continuing transformation of the design studio model. Fleischmann (2021) found that lectures and software training could move online, while project-based practice, face-to-face guidance, and immediate feedback remained in the studio. Yorgancıoğlu and Genel (2022) also noted that traditional and digital tools need to work together at different stages. These studies show that digitalisation did not eliminate embodied practice, but changed its position, form, and proportion within design learning.

After AI entered design learning, this transformation accelerated further. Generative AI can produce multiple visual alternatives within a short period, accelerating conceptual exploration, but it may also compress the process of developing ideas through gradual sketch revision (Fleischmann, 2025, 2026). Özorhon et al. (2025) found that students used existing designs as inputs for AI and then returned generated outcomes to the subsequent design process for further adjustment. This suggests that AI does not simply replace Embodied Hands-on. Instead, it reorganises the sequence and relationship between embodied practice and digital generation within the design process. Embodied Hands-on remains present, but increasingly alternates and connects with other forms of design activity.

### 3.2 AI iteration and cognitive hands-on

Generative AI has changed the way design iteration takes place. Zhu et al. (2025a) categorised students’ prompting behaviours as generation, modification, and selection, and found that the use of AI alone did not directly improve overall creativity, whereas multi-step operations were positively associated with the creativity of the final work. Zhu et al. (2025b) further noted that teachers could use the AI generation process to observe changes in design proposals and discuss the basis for students’ choices. Çiçek and Özkar (2025) found that AI could rapidly expand the range of design alternatives, but generated outcomes might lose direction in the absence of clear tasks and expert feedback. Together, these studies show that AI-supported design iteration is not a one-off act of generation, but consists of a sequence of activities including generation, selection, evaluation, and revision.

Language becomes an important operational medium in this process. Students need to translate design intentions into information that AI can process. Gökdemir and Kayan (2025) described AI design tools as a language interface, through which students need to express their design intentions and judge whether the generated outcomes respond to their original ideas. Karadağ and Ozar (2025) and Özorhon et al. (2025) also showed that students reorganise and redefine design problems while continuously adjusting prompts. Prompting is therefore not simply the input of keywords. It also becomes a way for students to organise ideas and advance the design process.

The continuous generation of AI outputs also changes when reflection occurs. Students need to repeatedly judge whether newly generated outcomes align with their design intentions rather than accepting AI outputs directly (Wang et al., 2025). Shin et al. (2026) found that students initially tended to expect AI to provide direct answers that matched their expectations. As their experience increased, they gradually became able to identify useful elements in incomplete or uncertain outputs and reconsider the function and context of the design. This suggests that the uncertainty of AI outputs can itself create opportunities for reflection.

Across these studies, prompting, generating, comparing, selecting, and revising repeatedly appear as interconnected learning activities. This review synthesises this pattern as Cognitive Hands-on. Unlike Embodied Hands-on, which relies primarily on direct making and material manipulation, Cognitive Hands-on develops through learners’ continued operation, judgement, and adjustment of AI-generated outcomes.

### 3.3 Emerging capabilities in AI-supported hands-on learning

As AI enters design learning, students need to integrate generated content with existing design knowledge, task requirements, and subsequent design processes. Studies by Özorhon et al. (2025), Gupta and Khan (2025), and Gül et al. (2024) show that AI can help students develop concepts and expand the scope of exploration, but generated outcomes usually still require further selection, adjustment, and development before they can become part of a complete design process. AI-supported design activities therefore involve more than tool use. They also require students to establish connections across different forms of information, tools, and stages of design.

This change first places greater demands on students’ integrative capabilities. AI can rapidly generate visual outcomes, but students still need to judge how these outputs should enter the subsequent design process. The teaching arrangements described by Özorhon et al. (2025) and Gökdemir and Kayan (2025) both required students to establish their own design concepts before introducing AI at a particular stage. Karadağ and Ozar (2025) also found that generated content needed further development before it could connect with the original design direction. Together, these studies show that one important capability in AI-supported learning is the ability to integrate generated outcomes with existing concepts, design stages, and other tools.

At the same time, the studies repeatedly highlight the importance of judgement and control over design direction. Students need not only to decide which generated outcomes should be retained, but also to judge which should be revised, discarded, or regenerated. As shown in Sections 3.1 and 3.2, AI-generated outcomes do not automatically determine the direction of the design. Students still need to make choices through continued comparison and revision. This process of judgement is closely connected with learner agency, particularly whether students can retain control over problem definition, outcome selection, and the final direction of the design when AI is involved.

Overall, the capabilities emerging in AI-supported environments do not constitute a single new skill. Rather, they involve a set of interconnected capabilities, including integration, judgement, and learner agency. As some acts of generation and revision shift toward human–AI interaction, the role of students does not diminish. Instead, it becomes increasingly visible in how they organise tools, evaluate outcomes, and maintain the direction of the design.

## 4 Discussion

### 4.1 The expansion and internal tensions of hands-on learning

This study shows that AI has not caused hands-on learning to disappear from design education. Rather, it has changed the ways in which learners engage in hands-on processes. Sketching, model making, and material experimentation continue to support ideation, feedback, and design refinement (Akalın and Sezal, 2009; Yorgancıoğlu and Genel, 2022). At the same time, prompt revision, comparison of generated outcomes, and human–AI iteration have begun to support some aspects of design exploration (Gökdemir and Kayan, 2025; Zhu et al., 2025a, b). These two forms of activity can be connected within the same design process and are not mutually exclusive (Öner et al., 2025; Özorhon et al., 2025). This study describes the former as Embodied Hands-on and the latter as Cognitive Hands-on. This distinction does not imply that all uses of AI constitute hands-on learning. The defining feature of Cognitive Hands-on is not the use of AI itself, but the learner’s continued engagement in generating, comparing, judging, and revising outcomes in response to feedback. AI-supported activity acquires the process characteristics of hands-on learning only when such participation is sustained.

This expansion can be understood through constructivism and experiential learning theory. Learners do not passively receive knowledge. They develop understanding through interaction with tasks and environments, accompanied by cycles of experience, reflection, conceptualisation, and further action (Kolb, 1984). From this perspective, both Embodied Hands-on and Cognitive Hands-on may involve action, feedback, reflection, and refinement, but they do not provide the same learning experience. Embodied Hands-on exposes students directly to material resistance and real-world conditions, whereas Cognitive Hands-on enables rapid comparison of alternatives and a wider scope of exploration. The two forms therefore share a process structure while relying on different sources of feedback and different learning conditions. This distinction is discussed further in the next section through embodied cognition theory.

AI-supported design exploration presents two possibilities: it may expand learning opportunities, but it may also compress learning processes. Rapid generation reduces the cost of testing design alternatives, while multiple rounds of generation, comparison, and revision may create new forms of iteration (Agboola and Yassin, 2025; Fleischmann, 2026; Zhu et al., 2025a, 2025b). At the same time, generation efficiency may compress exploratory processes previously carried out through sketching, materials, and gradual refinement, reducing opportunities for students to encounter failure and material constraints (Fleischmann, 2025, 2026). Such difficulties might otherwise encourage students to reinterpret problems and revise their judgements. An increase in the number of iterations enabled by AI therefore does not necessarily indicate an increase in iterative depth.

These possibilities should not be reduced to a binary claim that AI either strengthens or weakens hands-on learning. More important questions concern the forms of feedback provided by different activities, whether students remain involved in interpretation and revision, and which learning goals these processes support. AI-supported cognitive interaction may expand the forms of hands-on learning, but this does not demonstrate that Embodied Hands-on has lost its value. Instead, it reveals a new internal tension within hands-on learning: generation may become faster and feedback more abundant, while the depth of learning still depends on how students interpret, judge, and use that feedback.

Current understanding is also constrained by inconsistent definitions and measurement approaches across existing studies, as discussed further in Section 4.5. The conceptual distinction proposed in this study is therefore both a synthesis of the existing research and a response to these limitations. Embodied Hands-on and Cognitive Hands-on may both contribute to design learning, but there is currently insufficient evidence to show that they can replace one another. This study therefore proposes a conceptual framework for understanding how hands-on learning is expanding in the age of AI.

**Figure 4:**
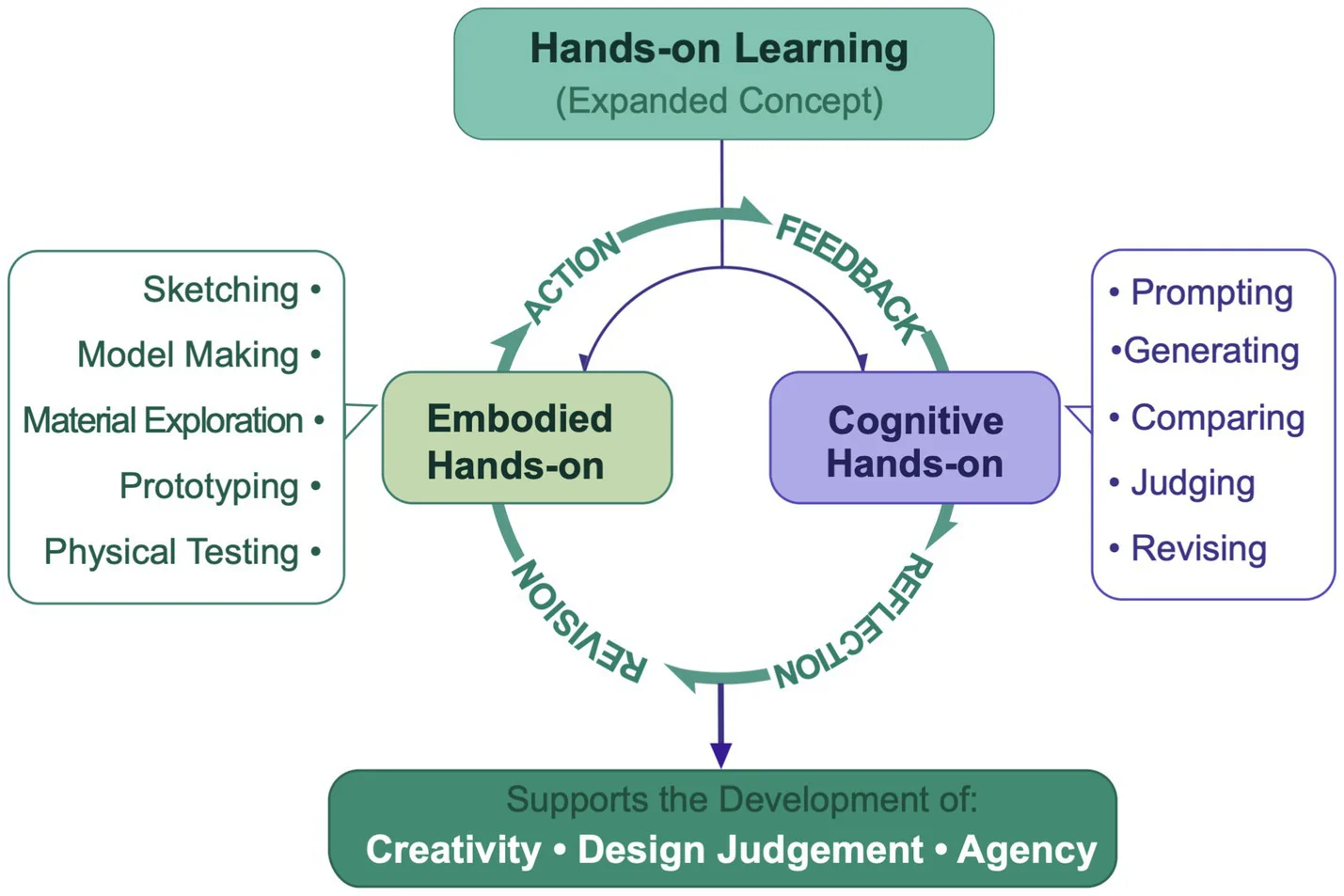
An expanded framework of hands-on learning.

Figure 4 presents hands-on learning in its expanded form as two interconnected components: Embodied Hands-on and Cognitive Hands-on. Both share a common cycle of action, feedback, reflection, and refinement while relying on different sources of feedback. Together, they support the development of creativity, design judgement, and student agency.

### 4.2 Why embodied hands-on remains irreplaceable

The conceptual expansion of hands-on learning does not mean that Embodied Hands-on has lost its role. Cognitive Hands-on mainly relies on language and visual outcomes as sources of feedback. Embodied Hands-on relies on bodily action, materials, and real-world conditions. The two forms therefore depend on different sources of feedback and should not be treated as educationally equivalent.

Embodied cognition theory suggests that cognition does not occur independently of the body (Wilson, 2002; Barsalou, 2008). Sketches and models do not simply represent ideas that have already been fully formed. Students often develop a clearer understanding of a problem through drawing, making, and observing. The weight, structure, and connections of materials can also constrain particular ideas and prompt students to revise their judgements.

Schön’s (1983) theory of reflective practice further explains that thinking through making is a key mechanism in the development of design thinking. When a model cannot stand, materials behave unexpectedly, or a prototype does not meet expectations, students need to reinterpret the problem. This reflection is embedded in the making process rather than occurring only afterwards. Material resistance and failure therefore provide important conditions for the development of judgement.

Embodied hands-on also provides a form of learning that AI cannot replace: it brings design back into reality. AI can generate visually convincing images, but appearing plausible does not mean that a design is practically feasible. Whether the materials work, whether the body can interact with the design, whether the scale is realistic, and whether the proposal responds to specific users still need to be tested through physical models and real-world conditions.

Retaining embodied hands-on is therefore not a rejection of AI. It preserves a learning mechanism that AI cannot replace. Current evidence does not show that Cognitive Hands-on can fully reproduce the educational role of Embodied Hands-on. Design education still needs to organise the relationship between the two forms deliberately according to different learning objectives.

### 4.3 Cognitive hands-on in design iteration

As AI takes on part of the design generation process, the ways in which students participate also change. Students are no longer involved only in direct drawing and making. They participate more in comparing outcomes, selecting alternatives, and developing designs further. Creative participation does not disappear. Instead, it shifts partly toward the interpretation, judgement, and further development of generated outcomes.

This change is consistent with the creative cognition framework proposed by Finke et al. (1992), in which creativity develops through movement between generation and exploration. AI can take on part of the generation stage, but exploration still requires students’ active involvement. Students need to identify which outcomes have potential, which deviate from the intended goal, and how the design should be developed further. Agboola and Yassin (2025) also noted that AI can expand idea generation and design exploration, but it cannot replace learners in establishing criteria, understanding context, and selecting directions. The educational value of Cognitive Hands-on therefore does not arise from AI generation itself, but from how learners continuously regulate the design process through comparison, judgement, selection, and revision. AI changes the distribution of tasks, but it does not remove students from the creative process.

This change also increases the importance of metacognition. Students need not only to judge AI-generated outcomes, but also to reflect on their own processes of judgement. They need to consider whether they have moved away from the original goal or accepted a visually complete outcome too early (Dwivedi et al., 2023; Kasneci et al., 2023). However, the increased emphasis on judgement may also create new limitations. When the design process depends more heavily on criteria and outcome comparison, learning may become more evaluation-oriented. This can reduce students’ willingness to retain unexpected outcomes and take aesthetic risks, while also narrowing the space for tolerance of uncertainty and trial and error.

Cognitive interaction therefore develops into a sustained form of Cognitive Hands-on only when judgement continues to lead to design revision and further action. Creativity is no longer expressed only through the generation of initial ideas. It is also expressed through how students select, reject, and further develop design alternatives.

### 4.4 Language expression in hands-on learning

Generative AI is gradually changing language from a tool for explaining design outcomes into an operational medium within the design process. Students need to transform vague ideas into clearer descriptions so that AI can generate designs accordingly. Language is therefore no longer used only to explain a completed design. It also begins to contribute to the advancement of the design process itself (Gökdemir and Kayan, 2025).

This change makes language expression an important part of Cognitive Hands-on. When students revise prompts, they are not only adjusting words. They are also redefining the direction of the design. AI outputs then become new sources of feedback, encouraging students to clarify their intentions and refine their descriptions further. Language expression therefore acquires the process characteristics of hands-on learning only when it is connected with generation, feedback, and revision.

This also creates new requirements for design learning. Students need more than general language skills. They also need to describe design intentions accurately. Studies by Karadağ and Ozar (2025) and Özorhon et al. (2025) show that students need to form a basic design direction before translating it into linguistic or visual information that can guide AI. Language ability is therefore connected with design judgement and process control.

Language-based interaction does not necessarily produce only positive effects. Some feelings, intuitions, and material experiences in design cannot easily be expressed fully in words. When students repeatedly organise design problems in ways that AI can recognise, they may become more inclined to develop proposals that are easier to describe and generate. Ideas that are difficult to express in language may be abandoned too early. This change may improve the clarity of design expression, but it may also limit open exploration and individual variation.

Design education should therefore not treat language ability simply as a skill in prompt writing. It is more important to help students understand how language shapes the direction of AI generation and which design experiences cannot depend entirely on verbal expression. Language can support the clarification of design intentions, but it cannot replace the other forms of feedback provided by Embodied Hands-on.

From the perspective of hands-on learning, language does not replace action. It becomes one of the new forms of action. It can connect design intentions, AI feedback, and subsequent revision, but its educational value still depends on whether students retain control over the direction of the design. Future design learning needs to consider not only whether students can express their ideas accurately, but also whether they can recognise the limits of language and return AI-generated outcomes to Embodied Hands-on for further testing.

### 4.5 Research contributions, limitations, and future research

The main contribution of this study lies in proposing an expanded concept of hands-on learning based on a thematic review. This concept distinguishes between Embodied Hands-on and Cognitive Hands-on. The distinction is not intended to create a binary opposition between traditional and emerging activities. It shows that hands-on learning is developing a more complex internal structure. The two forms depend on different sources of feedback, but they can connect and alternate within the same design process. Hands-on learning in the age of AI should therefore not be understood as a one-way transition from physical making to digital operation. It is better understood as a reorganisation of different forms of practice.

This framework also extends existing discussions of human–AI collaboration. Previous studies have commonly emphasised the role of AI in expanding idea generation and supporting human–AI collaboration (Agboola and Yassin, 2025; Agboola and Yassin, 2025). This study further argues that AI-enhanced design exploration does not automatically demonstrate that these two forms of hands-on activity have the same educational value. Expanding the range of generated possibilities and increasing the depth of learning are not the same issue.

Another contribution of this study is that it shifts the discussion from whether AI replaces hands-on learning to the educational goals supported by different forms of hands-on learning. Embodied Hands-on may more directly support real-world testing. Cognitive Hands-on may strengthen comparison and judgement.

This study has four main limitations. First, the proposed framework is conceptual and has not yet been empirically tested. There is still insufficient evidence to determine whether the two forms of activity can replace one another. Second, existing studies do not use a consistent definition of hands-on learning, and their measurement approaches also differ. These include assessments of final work, the number of generated outcomes, and interaction records. Such indicators may not fully reflect the quality of hands-on learning processes. Third, longitudinal evidence on the long-term educational effects of AI remains limited. Existing studies mainly examine performance within a single course or short-term project and therefore cannot fully explain how students’ capabilities may change as Cognitive Hands-on becomes more prominent over time. Fourth, the review relied primarily on Web of Science and Scopus. Although ERIC was explored during the initial scoping stage, the use of these two databases as the main sources of literature identification may have limited the comprehensiveness of the review and may have excluded relevant studies indexed elsewhere.

Future research could proceed in three directions. First, clearer operational definitions of hands-on learning could be established, together with methods for assessing the quality of action, feedback, reflection, and refinement rather than recording only the number of generated outcomes or the final work. Second, longitudinal or comparative studies could examine the different effects of Embodied Hands-on, AI-supported activities, and their combination on learning outcomes. Third, greater attention could be given to students’ own experiences, including how they understand the division of work between humans and AI, maintain control over the design process, and identify when AI supports or restricts personal expression.

Overall, this study does not argue that Cognitive Hands-on should replace Embodied Hands-on. It also does not argue that design education should reject AI. A more appropriate direction is to organise the two forms according to different learning objectives and further evaluate their respective educational roles. AI expands the conceptual boundaries of hands-on learning, but whether this expansion can preserve the depth of learning still depends on whether students remain involved in action, judgement, reflection, and refinement.

## 5 Conclusion

This study adopted a thematic review to examine how hands-on learning is changing in design education in the age of AI. The central finding is that AI has not eliminated hands-on learning. It has changed the conditions and forms through which it takes place. This transformation is reflected in two complementary strands: Embodied Hands-on and Cognitive Hands-on. Both share a cycle of action, feedback, reflection, and refinement, but they rely on different sources of feedback and should not be treated as directly interchangeable.

The main theoretical contribution of this study lies in proposing a process-oriented framework for understanding hands-on learning. Hands-on learning is conceptualised as a continuous cycle of action, feedback, reflection, and refinement rather than as a collection of specific making activities. This perspective shifts attention from whether AI tools are used to how the learning process itself is reconstructed. It also moves beyond the binary debate over whether AI replaces traditional making and instead asks what different forms of learning can contribute in different educational contexts.

From a pedagogical perspective, the findings suggest that design educators need to reconsider the balance between efficiency and depth. AI can increasingly accelerate idea generation and visual production, but this does not automatically lead to better learning. When educational processes become overly outcome-oriented, opportunities for iterative experimentation, critical reflection, and the development of creative agency may decrease. The most urgent challenge for design education is therefore not whether AI should enter the classroom, but how process-oriented learning can be deliberately supported within human–AI collaborative workflows.

This transformation also suggests that design literacy needs to place greater emphasis on evaluative judgement, metacognitive awareness, and learner agency in human–AI collaboration. Educational goals need to evolve accordingly. The current challenge is no longer only to teach students how to use AI tools. It is also to help them maintain awareness of their own reasoning, judgement, and creative processes when working with intelligent systems.

The limitations of this study and directions for future research have been discussed in detail in Section 4.5 and are not repeated here. Taken together, the findings point toward a complementary rather than competitive relationship between Cognitive Hands-on and Embodied Hands-on, with students’ involvement in action, judgement, reflection, and refinement remaining central to the educational value of both throughout human–AI collaboration.

## Data Availability

The original contributions presented in the study are included in the article/Supplementary material, further inquiries can be directed to the corresponding author.
