# Supplementary material for: Is hands-on learning still necessary in the age of AI? A thematic review
Source: Front Psychol. 2026 Sep 9;17:1897168. doi: 10.3389/fpsyg.2026.1897168 (PMC13597826; doi:10.3389/fpsyg.2026.1897168)
Supplement: Supplementary file 1 [file Data_Sheet_1.PDF]

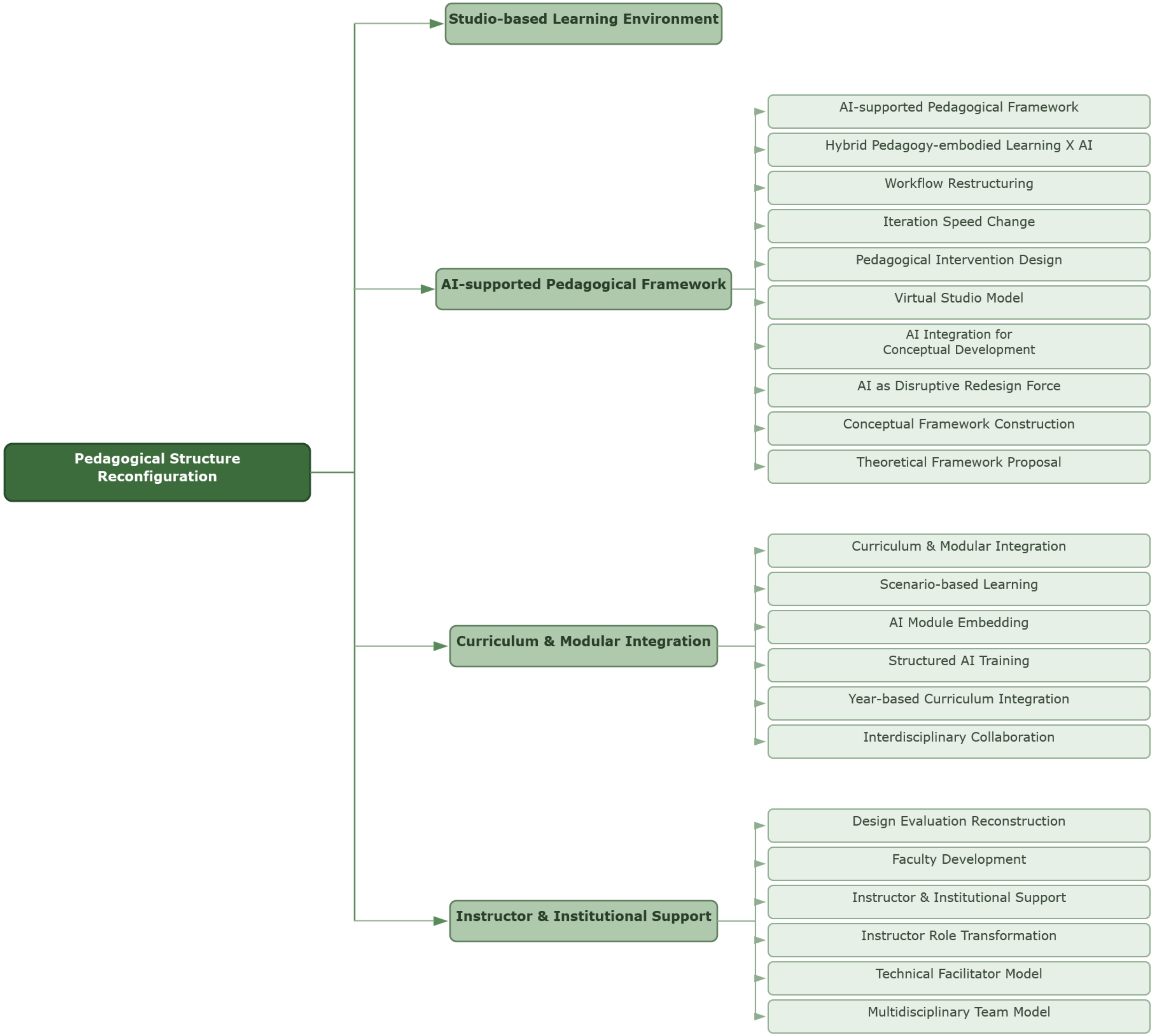

Pedagogical Structure Reconfiguration
Studio-based Learning Environment
AI-supported Pedagogical Framework
AI-supported Pedagogical Framework
Hybrid Pedagogy-embodied Learning X AI
Workflow Restructuring
Iteration Speed Change
Pedagogical Intervention Design
Virtual Studio Model
AI Integration for Conceptual Development
AI as Disruptive Redesign Force
Conceptual Framework Construction
Theoretical Framework Proposal
Curriculum & Modular Integration
Curriculum & Modular Integration
Scenario-based Learning
AI Module Embedding
Structured AI Training
Year-based Curriculum Integration
Interdisciplinary Collaboration
Instructor & Institutional Support
Design Evaluation Reconstruction
Faculty Development
Instructor & Institutional Support
Instructor Role Transformation
Technical Facilitator Model
Multidisciplinary Team Model

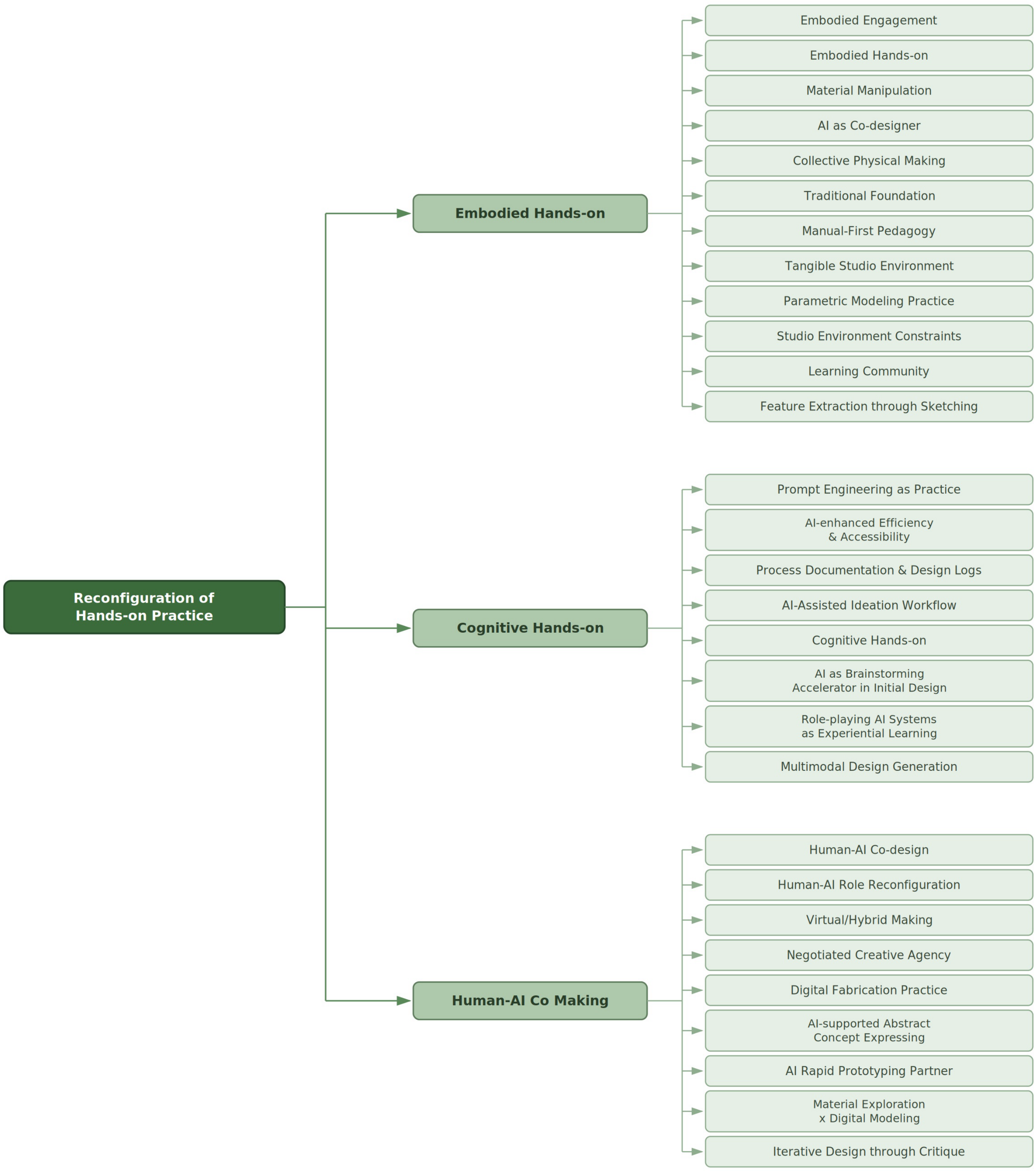

Reconfiguration of Hands-on Practice
Embodied Hands-on
Embodied Engagement
Embodied Hands-on
Material Manipulation
AI as Co-designer
Collective Physical Making
Traditional Foundation
Manual-First Pedagogy
Tangible Studio Environment
Parametric Modeling Practice
Studio Environment Constraints
Learning Community
Feature Extraction through Sketching
Cognitive Hands-on
Prompt Engineering as Practice
AI-enhanced Efficiency & Accessibility
Process Documentation & Design Logs
AI-Assisted Ideation Workflow
Cognitive Hands-on
AI as Brainstorming Accelerator in Initial Design
Role-playing AI Systems as Experiential Learning
Multimodal Design Generation
Human-AI Co Making
Human-AI Co-design
Human-AI Role Reconfiguration
Virtual/Hybrid Making
Negotiated Creative Agency
Digital Fabrication Practice
AI-supported Abstract Concept Expressing
AI Rapid Prototyping Partner
Material Exploration x Digital Modeling
Iterative Design through Critique

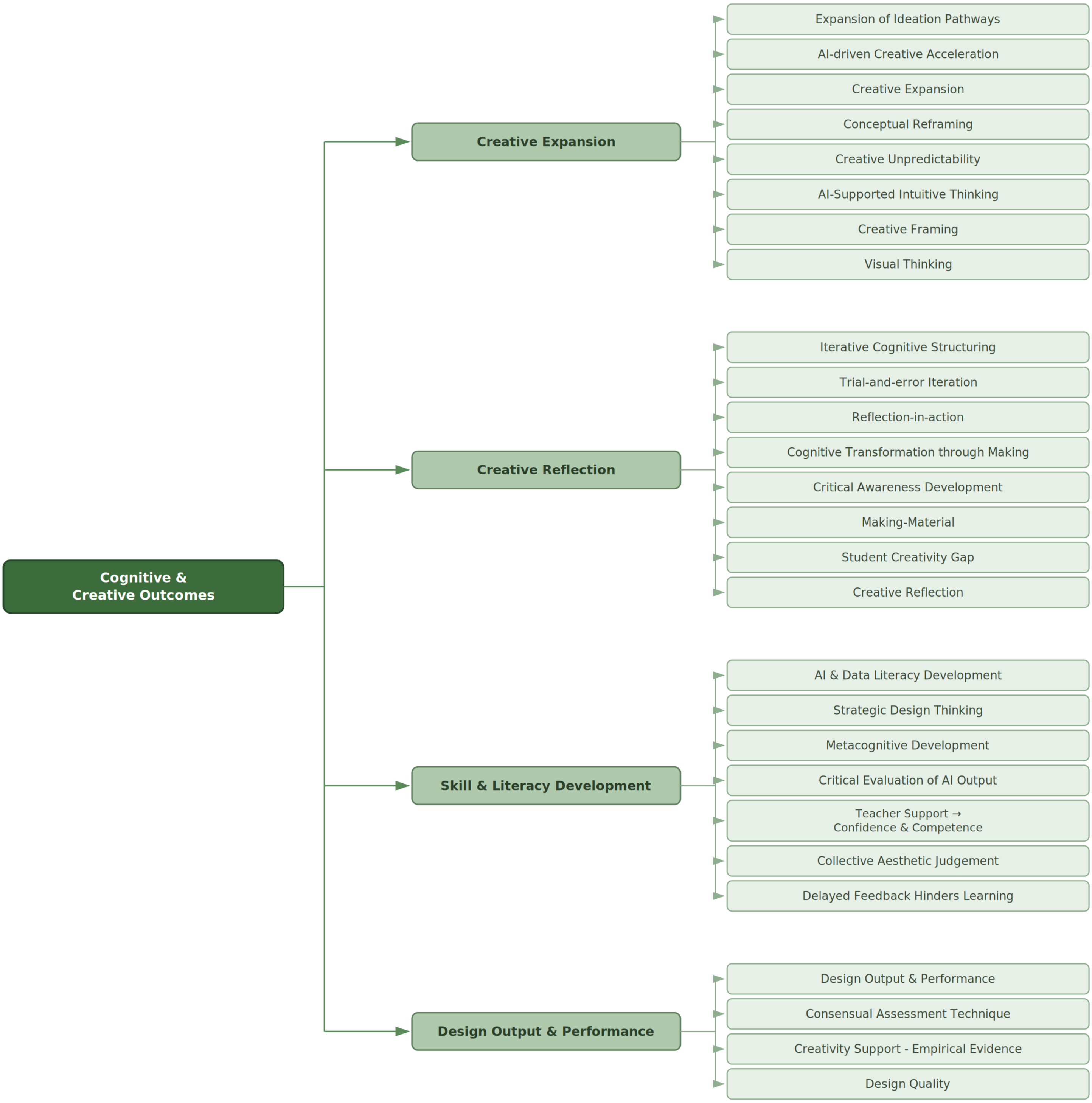
Cognitive & Creative Outcomes
Creative Expansion
Expansion of Ideation Pathways
AI-driven Creative Acceleration
Creative Expansion
Conceptual Reframing
Creative Unpredictability
AI-Supported Intuitive Thinking
Creative Framing
Visual Thinking
Creative Reflection
Iterative Cognitive Structuring
Trial-and-error Iteration
Reflection-in-action
Cognitive Transformation through Making
Critical Awareness Development
Making-Material
Student Creativity Gap
Creative Reflection
Skill & Literacy Development
AI & Data Literacy Development
Strategic Design Thinking
Metacognitive Development
Critical Evaluation of AI Output
Teacher Support → Confidence & Competence
Collective Aesthetic Judgement
Delayed Feedback Hinders Learning
Design Output & Performance
Design Output & Performance
Consensual Assessment Technique
Creativity Support - Empirical Evidence
Design Quality

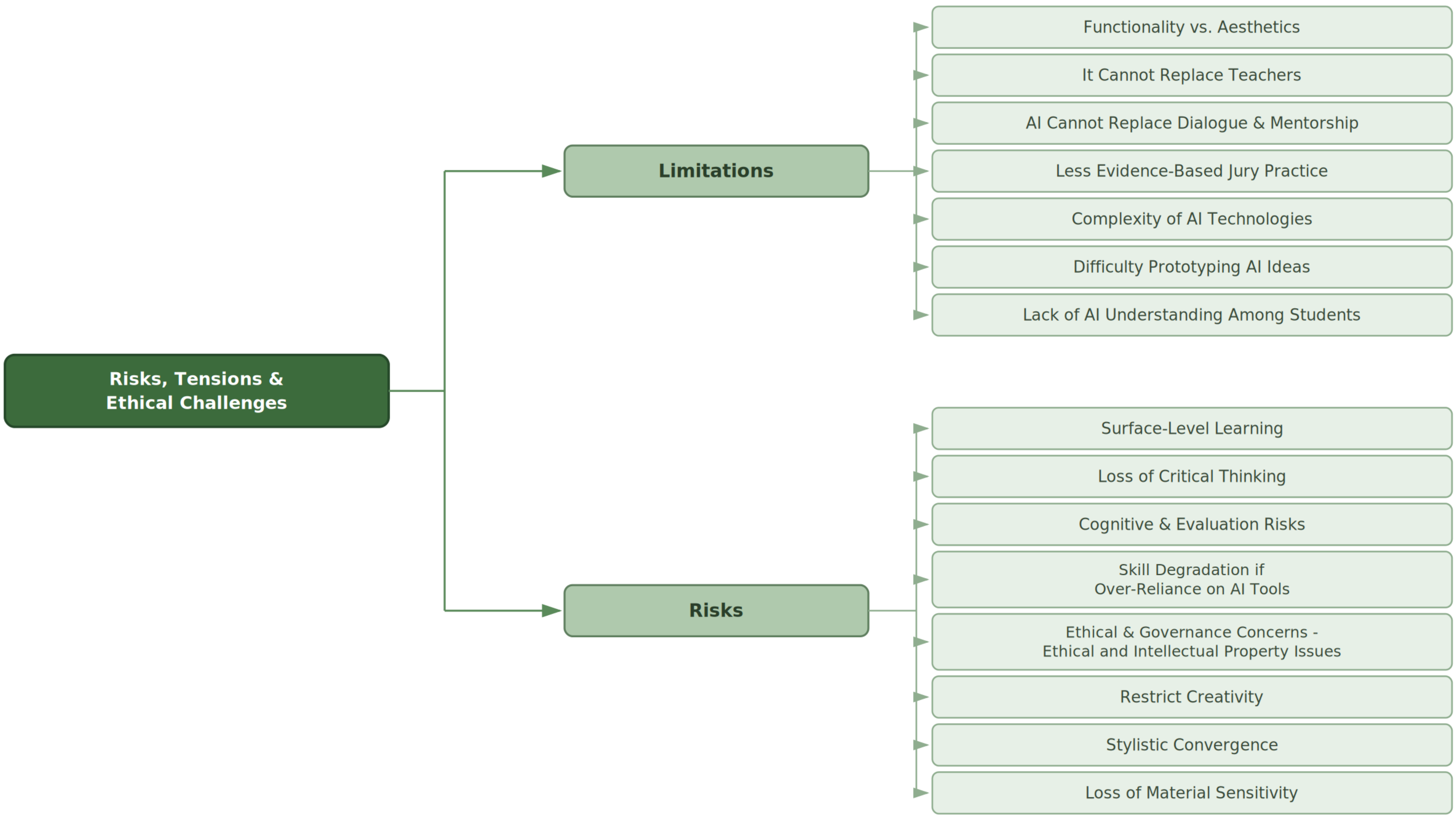
Risks, Tensions & Ethical Challenges
Limitations
Functionality vs. Aesthetics
It Cannot Replace Teachers
AI Cannot Replace Dialogue & Mentorship
Less Evidence-Based Jury Practice
Complexity of AI Technologies
Difficulty Prototyping AI Ideas
Lack of AI Understanding Among Students
Risks
Surface-Level Learning
Loss of Critical Thinking
Cognitive & Evaluation Risks
Skill Degradation if Over-Reliance on AI Tools
Ethical & Governance Concerns - Ethical and Intellectual Property Issues
Restrict Creativity
Stylistic Convergence
Loss of Material Sensitivity
